# Improving Access to Clinical Guidelines Through a Centralised Digital Platform in an NHS Setting: A Quality Improvement Study

**DOI:** 10.7759/cureus.118021

**Published:** 2026-10-16

**Authors:** Tanushree Dewan, Akhil Tomy, Oladapo Adesua, Apurva Bharucha, Sakkarai Ambalavanan

**Affiliations:** 1 Internal Medicine, The Royal Free Hospital, London, GBR; 2 Internal Medicine, Betsi Cadwaladr University Health Board, Rhyl, GBR; 3 Internal Medicine, Betsi Cadwaladr University Health Board, Bodelwyddan, GBR; 4 Cardiology, Betsi Cadwaladr University Health Board, Rhyl, GBR; 5 Respiratory Medicine, Betsi Cadwaladr University Health Board, Rhyl, GBR

**Keywords:** clinical informatics, healthcare digitisation, patient safety, quality improvement, standardised care, workflow efficiency

## Abstract

Background

A recurring issue was identified among resident doctors at Glan Clwyd Hospital regarding difficulty locating clinical guidelines, which may impact decision-making and patient safety. Timely access to guidelines is particularly critical during out-of-hours shifts to ensure safe, evidence-based care.

Aim

To develop and implement a centralised SharePoint^TM^-based clinical guideline repository to improve the speed, ease, and efficiency of accessing clinical guidelines, and to evaluate its impact on staff experience.

Methods

A single-centre survey-based quality improvement project was conducted using independent pre- and post-intervention surveys within a Plan-Do-Study-Act (PDSA) framework. A baseline survey of clinical staff identified key barriers in accessing guidelines. A centralised SharePoint^TM^ site was then created, organising guidelines by speciality and clinical scenario. Training and communication were provided, followed by a post-intervention survey conducted.

Results

The pre-intervention survey had 80 respondents. Pre-intervention surveys showed the majority of clinicians found accessing guidelines via BetsiNet difficult, with n=59 (74%) reporting perceived delays to patient care and strong demand for an alternative platform. The post-intervention survey had 50 respondents. Following SharePoint^TM^ implementation, n=47 (94%) respondents reported faster access with perceived improvements in patient care and safety across grades. The platform accrued 764 page views over a 90-day period and 3,869 cumulative views since launch, demonstrating sustained engagement across all clinical grades.

Conclusion

The implementation of a centralised SharePoint^TM^ platform was associated with perceived improvements in timely access to clinical guidelines and enhanced staff confidence. This low-cost, scalable intervention utilises existing NHS digital infrastructure, supports efficient patient care via desktop and mobile device access, improves staff satisfaction and is readily replicable across other departments and health boards.

## Introduction

Access to up-to-date clinical guidelines is essential for safe and standardised patient care, particularly during on-call and out-of-hours periods when clinicians make decisions under significant time pressures. National patient safety priorities have highlighted that delays or gaps in current clinical information being readily available lead to inconsistent care quality and preventable risks [1]. Literature from America, Japan, China, the Middle East and North Africa shows that fragmented or outdated clinical resources lead to inconsistent care and reduced adherence to evidence-based practices. In fast-paced environments, delays in accessing important information negatively impact patient outcomes, workflow, and staff morale [2-5]. The NHS places strong emphasis on dependable information systems, as shown by frameworks that promote professional standards, drive digital transformation, and support ongoing quality improvement [6,7].

Although many guidelines are available, NHS clinicians often struggle to quickly find documents because of fragmented storage systems, outdated file paths and inconsistent local organisation. The NHS Records Management Code of Practice highlights the importance of clear and structured digital document management systems for easy retrieval and to avoid informal workarounds [8]. Poorly organised repositories slow clinical decisions, increase cognitive burden and delay patient care. This challenge is recognised in national quality-improvement guidance as a system issue that can be improved [9].

This challenge is particularly pronounced in North Wales, where Betsi Cadwaladr University Health Board (BCUHB) is currently at an early stage of digitisation. BCUHB does not yet have comprehensive electronic prescribing and clinical documentation systems and continues to rely predominantly on paper-based processes. This reflects the broader UK landscape, in which health boards and trusts vary considerably in their progress towards full digitisation, with the transition to electronic systems remaining an ongoing process for many organisations across the UK. In a predominantly paper-based environment, delivering comprehensive, centralised clinical decision support is inherently more difficult. Organisations with fully digital infrastructure are often able to embed decision support directly within their core systems, whereas BCUHB's current paper-based environment limits its ability to do so. Within this context, a low-cost, readily implementable platform such as SharePoint^TM^ offers a pragmatic means of improving workflow efficiency and guideline accessibility while broader digitisation efforts continue.

Digital platforms, such as SharePoint^TM^, are increasingly utilised within NHS environments for their ability to provide centralised, searchable, and securely managed repositories for operational and clinical documentation [10,11]. This strategy is consistent with NICE recommendations advocating for digital technologies that optimise workflow efficiency and facilitate access to evidence-based guidelines [12].

This project developed from firsthand experience of resident doctors during their out-of-hours shifts, who noted inconsistencies and time lost locating relevant policies and protocols. Guidelines were dispersed across multiple intranet locations, local desktops and paper formats, with successful retrieval dependent on prior knowledge of file structures or informal peer guidance. Following recognition of this gap, a quality improvement project was designed to address it. A centralised SharePoint^TM^ site was developed and implemented to streamline guideline access, simplify navigation and improve staff confidence. This study evaluates the impact of this intervention on staff experience and efficiency in retrieving clinical guidelines.

## Materials and methods

This quality improvement project was conducted in the general medical department at Glan Clwyd Hospital, part of BCUHB in the UK. The hospital is a district general hospital with a wide range of medical specialities, managing both acute and chronic medical admissions.

We used a pre- and post-intervention survey methodology as part of a Plan-Do-Study-Act (PDSA) cycle, allowing for iterative feedback and real-time refinement of the intervention in a single-centre study.

A structured survey was distributed electronically to 90 eligible resident doctors and physician associates within the general medicine department. The electronic surveys were circulated to eligible clinical staff using email distribution lists and official messaging groups. Participation was voluntary and anonymous. Eligible participants were resident doctors and physician associates working within the department, while consultants were excluded from the survey analysis. All survey data were collected anonymously in accordance with BCUHB information governance policies. The full pre-intervention survey questions are provided in Supplementary File 1.

The intervention comprised the design and implementation of a centralised SharePoint^TM^ -based clinical guideline repository for the general medicine department at Glan Clwyd Hospital. The SharePoint^TM^ site was designed with input from multidisciplinary clinical staff, including trainees, senior clinicians, and digital support. The rationale for selecting SharePoint^TM^ over the existing intranet was multifaceted. BetsiNet, the hospital intranet, could only be accessed on trust devices and was not available on personal or mobile devices, limiting point-of-care accessibility. SharePoint^TM^ offered greater ease of access via desktop and mobile devices compared to the fragmented BetsiNet system. Unlike the intranet, which competed with other organisational priorities for maintenance and updates, SharePoint^TM^ could be dedicated solely to clinical guideline management. It provided enhanced decision support through a structured, searchable interface, with robust content management tools enabling straightforward updating and version control. Crucially, the peer design and development of the platform ensured the end product reflected frontline needs. Finally, SharePoint^TM^ offered transparent editorial controls, allowing guideline owners to maintain direct oversight of content compared to the existing intranet. Furthermore, as SharePoint^TM^ is already integrated within the NHS Wales digital infrastructure, implementation required no bespoke software development or additional cost. Key features of the site included: a folder structure organised by speciality; search bar functionality; and a mobile-friendly layout.

The site structure was refined through iterative feedback from a small user group of consultant physicians. Once finalised, the platform was launched hospital-wide. Communication and awareness were supported through hospital grand rounds, email bulletins, and posters, as shown in Supplementary File 2.

After two months of use, a post-intervention survey was distributed electronically to staff. Both quantitative and qualitative questions were included. The full post-intervention survey questions are provided in Supplementary File 3.

Survey responses were analysed using frequencies (n) and percentages (%), as described in the Results section. As participants were anonymous and individual respondents could not be paired across pre- and post-intervention surveys, the two samples were independently analysed. SharePoint^TM^ analytics were summarised descriptively using page-view data.

Patients and members of the public were not involved in the design, conduct, or reporting of this quality improvement study. The study focused on healthcare professional workflows and system implementation. Results were disseminated to staff through departmental meetings.

## Results

Pre-intervention findings

The pre-intervention survey included 80 respondents. The pre-survey highlighted significant difficulties in accessing clinical guidelines via the existing BetsiNet system. With n=49 (61.3%) of respondents rating access as difficult or impossible, and only n=3 (3.75%) finding the process very easy (Figure 1a). Of respondents, n=59 (74%) perceived guideline inaccessibility delayed patient care, with a further n=20 (25%) uncertain (Figure 1b). n=72 (90%) reported that access to guidelines through a different mobile application would be helpful (Figure 1C). Respondents represented multiple clinical grades (Figure 1d).

**Figure 1:**
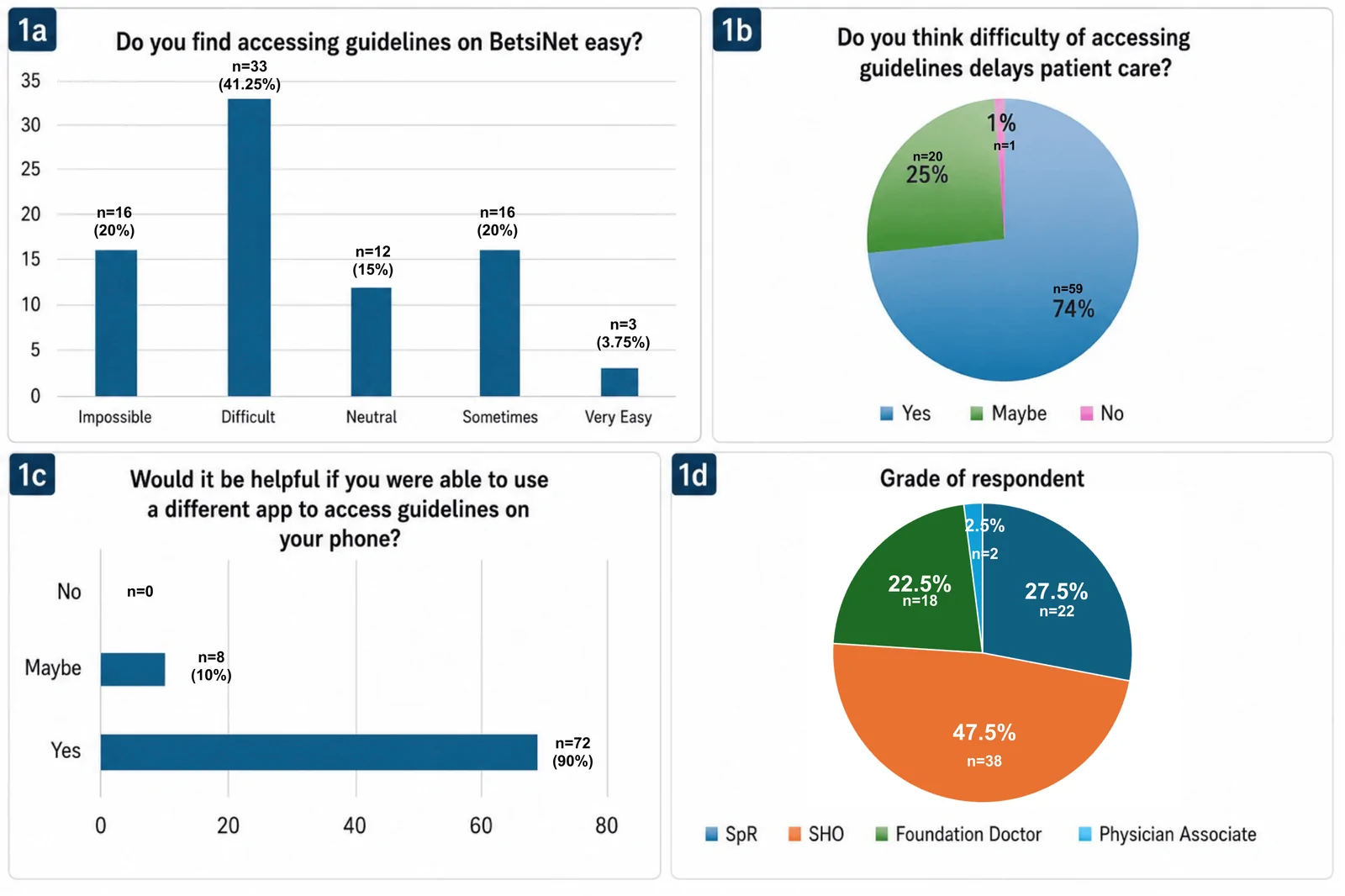
Pre-intervention survey results (n=80) 1A: Ease of accessing clinical guidelines on BetsiNet. 1B: Delays in patient care due to the accessibility of guidelines. 1C: Demand for app-based access to guidelines. 1D: Grade of survey respondents.

Post-intervention findings

The post-intervention survey included 50 respondents. Post-intervention survey results indicate an enhancement in user experience following the implementation of the SharePoint^TM^ guideline platform. n=47 (94%) of respondents agreed or strongly agreed that SharePoint^TM^ enabled faster access to clinical guidelines (Figure 2a). n=43 (86%) reported perceived improvement in patient care (Figure 2b), and n=45 (90%) reported perceived improved patient safety (Figure 2c). Disagreement was low across the assessed domains, ranging from n=1 (2%) to n= 2 (4%). Respondents represented multiple clinical grades (Figure 2d). Sustained platform engagement was evidenced by SharePoint^TM^ analytics, which recorded 764 site visits between 7 November 2025 and 4 February 2026, with 3,869 cumulative site visits recorded from platform launch in June 2025 (Figure 3).

**Figure 2:**
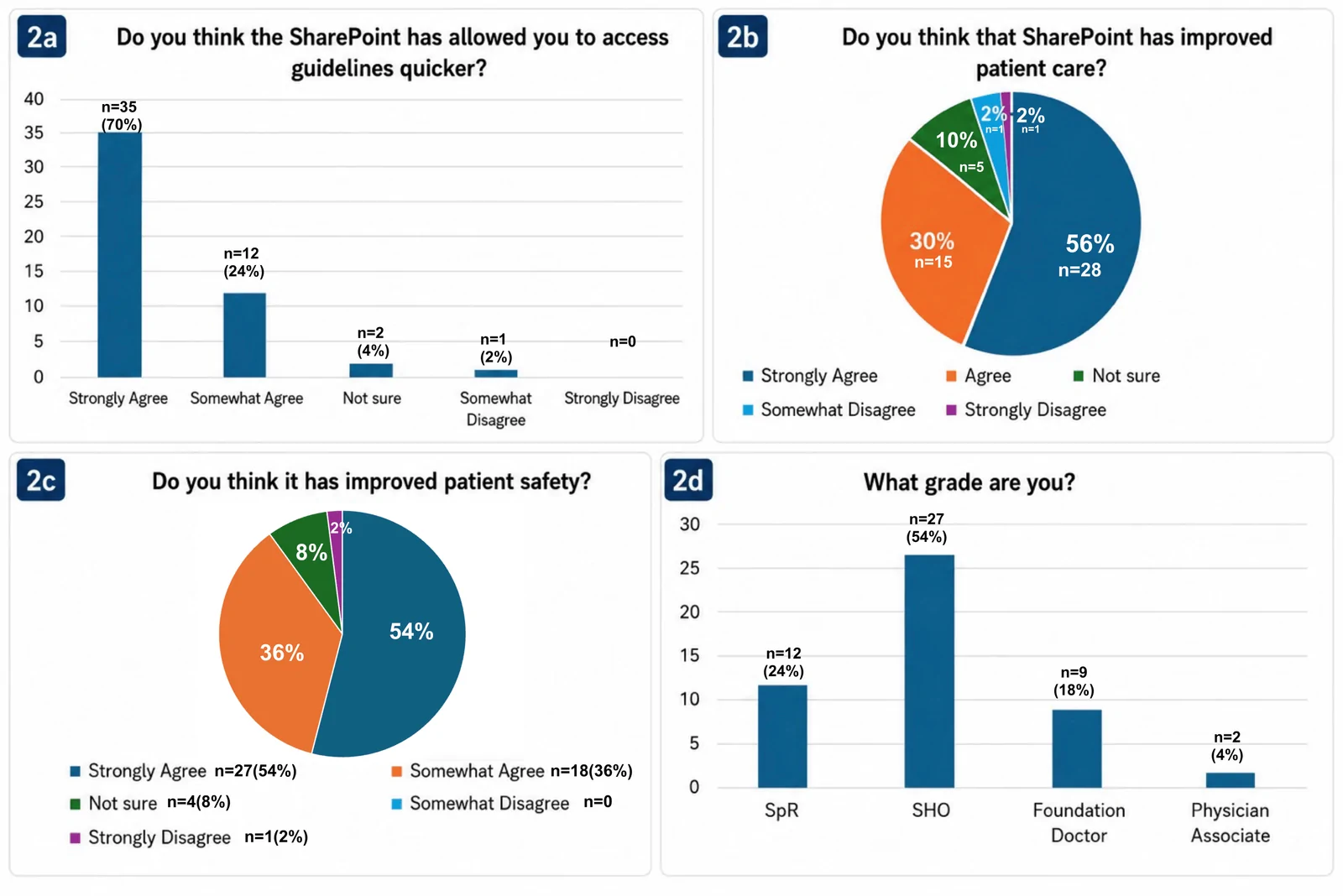
Post-intervention survey results (n=50) 2A: Improved access to guidelines via SharePoint. 2B: Perceived improvement in patient care. 2C: Perceived improvement in patient safety. 2D: Grade of survey respondents.

**Figure 3:**
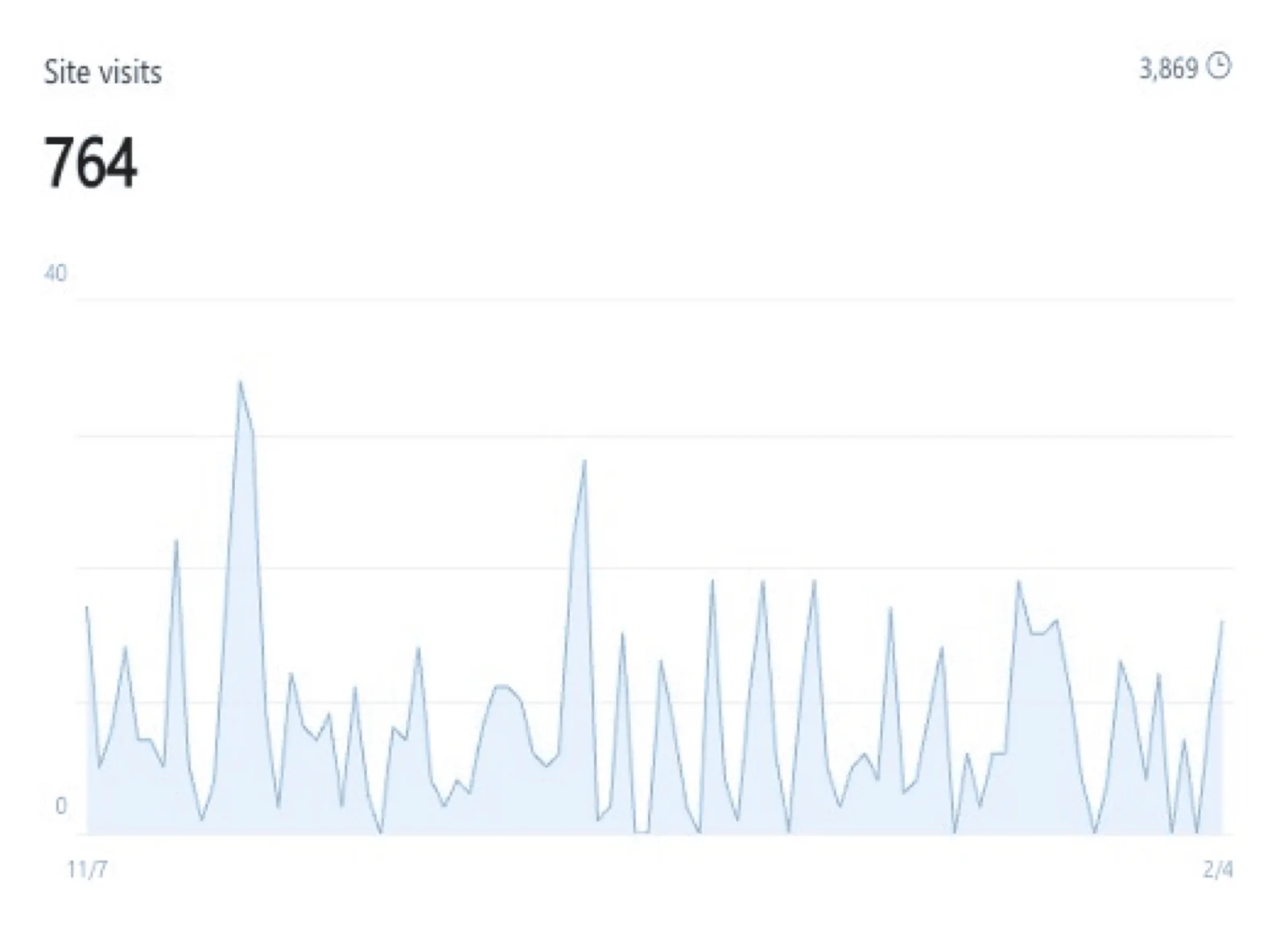
SharePoint site visit analytics showing 764 site visits between November 2025 and February 2026, and 3,869 cumulative site visits from platform launch in June 2025

## Discussion

This quality improvement project demonstrates a clear association between the introduction of a structured SharePoint-based guideline repository and improved accessibility of clinical guidelines, with perceived downstream benefits for patient care and safety.

Pre-intervention findings

The pre-survey highlighted significant difficulties in accessing clinical guidelines via the existing BetsiNet system. Among the 80 pre-intervention respondents, n=49 (61.3%) of respondents rated access as difficult or impossible (Figure 1a); the existing system did not adequately support timely access to essential clinical information during routine or acute clinical care. n=59 (74%) of respondents perceived that guideline inaccessibility delayed patient care (Figure 1b), reinforcing the link between digital infrastructure and clinical efficiency, suggesting poor accessibility may contribute to delays in decision-making, escalation, or initiation of evidence-based management. The strong demand for a mobile alternative, reported by n=72 (90%) respondents (Figure 1c), reflects the point-of-care needs of junior doctors working in fast-paced clinical environments. Respondents represented multiple clinical grades (Figure 1d), indicating that difficulties with guideline access were reported across different levels of seniority.

Post-intervention findings

Post-intervention survey results indicate an enhancement in user experience following the implementation of the SharePoint^TM^ guideline platform. Among the 50 post-intervention respondents, n=47 (94%) of respondents agreed or strongly agreed that SharePoint^TM^ enabled faster access to clinical guidelines (Figure 2a), effectively addressing the primary concern identified in the baseline survey. Perceived impact extended beyond usability alone, with n=43 (86%) reporting a perceived improvement in patient care (Figure 2b) and n=45 (90%) reporting a perceived improved patient safety (Figure 2c). These findings suggest that improved guideline accessibility may facilitate more timely, standardised, and evidence-based clinical decisions, particularly in acute or out-of-hours settings. Levels of disagreement across all domains were minimal, indicating broad acceptability of the platform across different clinician grades (Figure 2d). The post-survey respondents were drawn from the original pre-intervention respondent cohort, permitting group-level comparison between the two survey periods, although individual responses could not be matched.

Comparison with other digital guideline access initiatives

Similar quality improvement initiatives in other healthcare organisations have focused on improving clinicians’ access to clinical guidelines through centralised digital resources. The ‘Survive On Call’ project described by Penders and Mallet implemented a smartphone-based platform that consolidated hospital guidelines, referral pathways, and key operational information for doctors working out of hours. Following implementation, clinicians reported improved satisfaction with access to guidelines and reduced time spent locating essential information, supporting more efficient decision-making during acute clinical care [10]. Similarly, Clare et al. redesigned a hospital induction application at University Hospital Wishaw to streamline access to clinical protocols and organisational guidance for incoming doctors [11]. This intervention improved accessibility of local policies and enhanced clinicians’ confidence in unfamiliar clinical environments. A further quality improvement project described the redesign of the intranet page to improve user satisfaction and accessibility [13]. Together, these studies highlight that consolidating fragmented clinical information into a single, easily accessible digital platform is an effective strategy for improving usability and supporting adherence to evidence-based practice in hospital settings [10,11,13].

When compared with these initiatives, the SharePoint^TM^ -based repository project demonstrates similar benefits while offering several practical advantages within the NHS digital ecosystem. The centralisation of guidelines into a structured, searchable interface may reduce the cognitive and practical burden associated with locating clinical information and improve perceived efficiency of guideline retrieval among clinicians. However, some previously reported interventions utilised dedicated mobile applications that may provide additional features such as offline access, push notifications, and optimised point-of-care usability. In contrast, the SharePoint^TM^ solution prioritises accessibility, scalability, and ease of implementation by leveraging existing NHS digital infrastructure without the need for bespoke software development. Evidence from digital health implementation studies suggests that interventions integrated within existing organisational systems are often more sustainable and easier to maintain over time, particularly in resource-constrained healthcare environments [14,15].

Interpretation and implications

Collectively, these findings suggest that improving digital access to local clinical guidelines is associated with perceived improvements in efficiency, patient care, and safety. While the outcomes measured are subjective, they reflect real-world clinician experience and are highly relevant to frontline practice. The intervention aligns with NHS priorities around digital transformation, patient safety, and reduction of unwarranted variation in care [6,7].

The strong uptake and positive feedback support the acceptability and continued engagement with the SharePoint^TM^ platform and provide justification for further development, including expansion of guideline content, optimisation for mobile use, and integration with induction and teaching programmes.

Limitations

While conducting the quality improvement project, a few limitations were noted. The most significant was that the post-intervention survey coincided with trainee changeover. During this period, many colleagues were transitioning to new posts or moving to different hospitals, which likely contributed to a reduced response rate and may have affected the composition of the post-intervention data. Although all eligible clinicians were invited to participate, participation was voluntary, introducing the possibility of nonresponse and self-selection bias. The 50 post-intervention respondents came from the original cohort; anonymous surveys prevented individual matching. Therefore, changes were assessed at the group level only.

Additionally, the scope of this project was to evaluate whether the platform improved guideline accessibility and staff experience. Objective clinical outcomes and patient-level safety indicators were therefore not assessed. Future iterations may expand the evaluation to include these broader quality indicators to further validate the platform's impact.

Scope for expansion

Further development will be informed by additional PDSA cycles to ensure ongoing integration of user feedback. Next steps also include regular maintenance of the SharePoint^TM ^site to ensure that all guidance remains current and accurate. Future work will explore incorporating clinical outcome measures into the evaluation to better understand the platform’s impact on patient care and safety.

## Conclusions

The implementation of a centralised SharePoint^TM^-based guideline repository was associated with improved clinician-reported access to clinical guidelines, with n=47 (94%) of respondents reporting faster access and strong perceived benefits for patient care and safety across all grades. This low-cost intervention leverages existing NHS digital infrastructure, requiring no bespoke software development, and is potentially replicable across other departments and health boards with comparable digital infrastructure. The strong uptake and sustained platform engagement support its long-term viability. Future efforts will focus on expanding guideline content, optimising mobile accessibility, and incorporating objective clinical outcome measures to further evaluate the platform’s impact on patient care and safety.

## Figures and Tables

**Figure 4:**
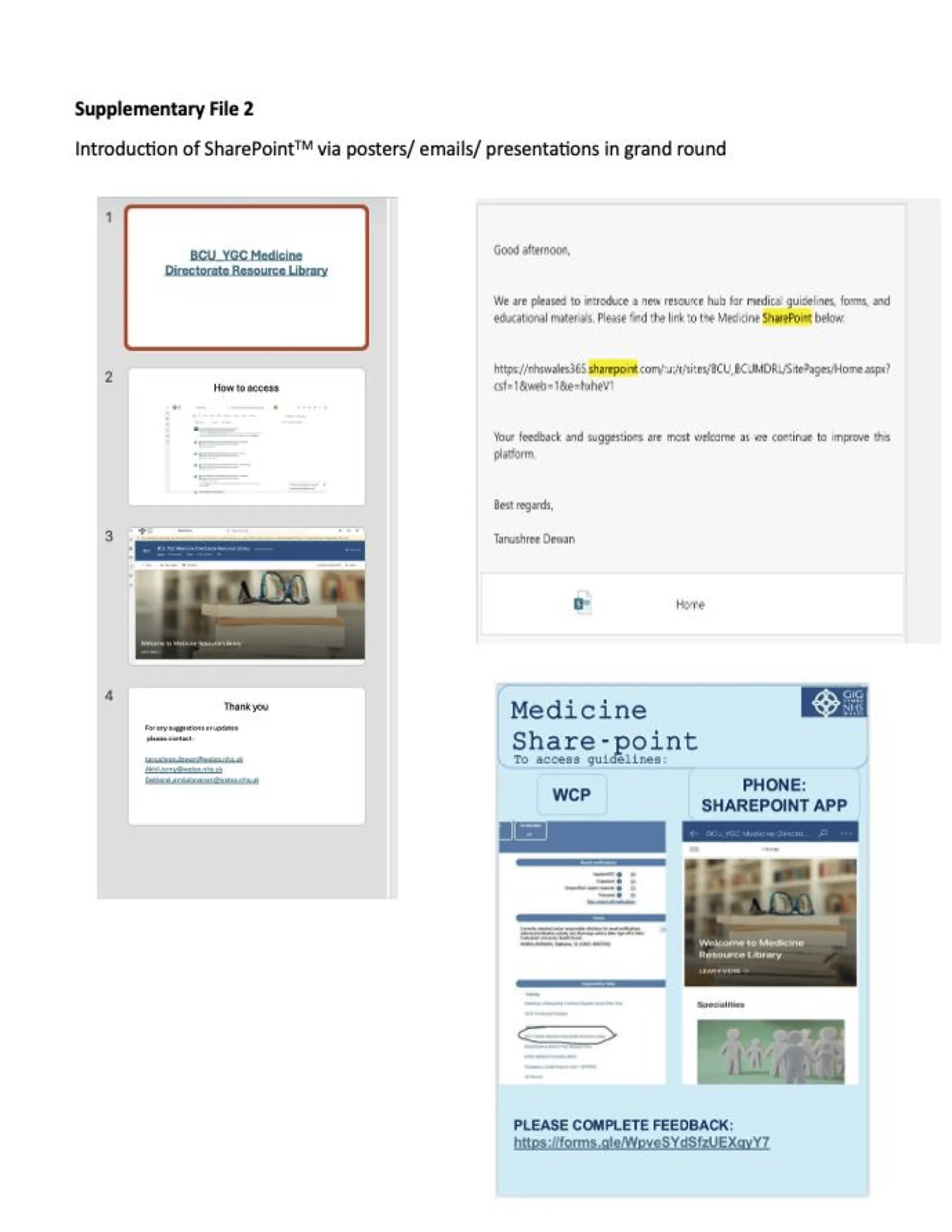
Introduction of SharePoint™ via posters/emails/presentations in the grand round

## Appendices

Supplementary file 1

Baseline Survey:

Question 1: Do you find accessing guidelines on BetsiNet easy?

a. Yes, very easy

b. Sometimes

c. Neutral

d. Difficult

e. Impossible

Question 2: Do you think difficulty of accessing guidelines delays patient care?

a. Yes

b. Maybe 

c. No

Question 3: Would it be helpful if you were able to use a different app to access guidelines on your phone?

a. Yes

b. Maybe

c. No

Question 4: What grade are you?

a. SpR 

b. SHO 

c. Foundation Doctor 

d. Physician Associate 

Supplementary file 2

Supplementary file 3

Post SharePoint^TM^ survey:

Question 1: Do you think the SharePoint has allowed you to access guidelines quicker?

a. Strongly Agree 

b. Somewhat Agree

c. Not Sure 

d. Somewhat Disagree 

e. Strongly Disagree

Question 2: Do you think that SharePoint has improved patient care?

a. Strongly Agree 

b. Agree

c. Not Sure 

d. Somewhat Disagree 

e. Strongly Disagree

Question 3: Do you think it has improved patient safety?

a. Strongly Agree 

b. Somewhat Agree

c. Not Sure 

d. Somewhat Disagree 

e. Strongly Disagree

Question 4: Are the guidelines up-to-date?

a. Yes

b. No

Question 5: Any guidelines that you would like to add to SharePoint?

Question 6: What grade are you?

a. SpR

b. SHO

c. Foundation Doctor

d. Physician Associate

## References

[1] (2019). NHS England. The NHS patient safety strategy. Safer culture, safer systems, safer patients. https://www.england.nhs.uk/patient-safety/the-nhs-patient-safety-strategy/.

[2] Qumseya B, Goddard A, Qumseya A, Estores D, Draganov PV, Forsmark C (2021). Barriers to clinical practice guideline implementation among physicians: a physician survey. Int J Gen Med.

[3] Sasaki N, Yamaguchi N, Okumura A (2020). Factors affecting the use of clinical practice guidelines by hospital physicians: the interplay of IT infrastructure and physician attitudes. Implement Sci.

[4] Jin Y, Li Z, Han F (2019). Barriers and enablers for the implementation of clinical practice guidelines in China: a mixed-method study. BMJ Open.

[5] Almazrou SH, Alfaifi SI, Alfaifi SH, Hakami LE, Al-Aqeel SA (2020). Barriers to and facilitators of adherence to clinical practice guidelines in the Middle East and North Africa region: a systematic review. Healthcare (Basel).

[6] (2023). NHS England. Professional standards: framework for quality assurance and improvement. https://www.england.nhs.uk/publication/professional-standards/.

[7] (2023). NHS England. Digital transformation. https://www.england.nhs.uk/digitaltechnology/.

[8] (2021). NHS England. Records Management Code of Practice for Health and Social Care. Care.

[9] (2017). NHS. Quality improvement: theory and practice in healthcare. https://www.england.nhs.uk/improvement-hub/wp-content/uploads/sites/44/2017/11/Quality-Improvement-Theory-and-Practice-in-Healthcare.pdf.

[10] Penders R, Mallet M (2016). Survive on call - a QI project to improve access to hospital clinical guidelines. BMJ Qual Improv Rep.

[11] Clare K, Cathcart J, Patel M (2020). Creating more than a directory: improving handover of information by renovating the induction app for University Hospital Wishaw. BMJ Open Qual.

[12] (2022). National Institute for Health and Care Excellence. Evidence standards framework for digital health technologies. London: NICE. https://www.nice.org.uk/corporate/ecd7.

[13] Walkden G, Millwood S, Cavanagh A (2015). Improving access to clinical guidance: redevelopment of a junior doctor intranet page. BMJ Qual Improv Rep.

[14] Wallace S, Clark M, White J (2012). 'It's on my iPhone': attitudes to the use of mobile computing devices in medical education, a mixed-methods study. BMJ Open.

[15] Gagnon MP, Ghandour el K, Talla PK (2014). Electronic health record acceptance by physicians: testing an integrated theoretical model. J Biomed Inform.

